# New Tissue-Engineered Vascular Matrix Based on Regenerated Silk Fibroin: *in vitro* Study

**DOI:** 10.17691/stm2023.15.4.04

**Published:** 2023-07-28

**Authors:** E.S. Prokudina, E.A. Senokosova, L.V. Antonova, E.O. Krivkina, E.A. Velikanova, T.N. Akentieva, T.V. Glushkova, V.G. Matveeva, N.A. Kochergin

**Affiliations:** Researcher, Laboratory of Tissue Engineering and Intravascular Visualization; Research Institute for Complex Issues in Cardiovascular Diseases, 6 Sosnovy Blvd., Kemerovo, 650002, Russia; Researcher, Laboratory of Tissue Engineering and Intravascular Visualization; Research Institute for Complex Issues in Cardiovascular Diseases, 6 Sosnovy Blvd., Kemerovo, 650002, Russia; Leading Researcher, Laboratory of Tissue Engineering and Intravascular Visualization; Research Institute for Complex Issues in Cardiovascular Diseases, 6 Sosnovy Blvd., Kemerovo, 650002, Russia; Junior Researcher, Laboratory for Cell Technology; Research Institute for Complex Issues in Cardiovascular Diseases, 6 Sosnovy Blvd., Kemerovo, 650002, Russia; Researcher, Laboratory for Cell Technology; Research Institute for Complex Issues in Cardiovascular Diseases, 6 Sosnovy Blvd., Kemerovo, 650002, Russia; Junior Researcher, Laboratory of New Biomaterials; Research Institute for Complex Issues in Cardiovascular Diseases, 6 Sosnovy Blvd., Kemerovo, 650002, Russia; Senior Researcher, Laboratory of New Biomaterials; Research Institute for Complex Issues in Cardiovascular Diseases, 6 Sosnovy Blvd., Kemerovo, 650002, Russia; Senior Researcher, Laboratory for Cell Technology; Research Institute for Complex Issues in Cardiovascular Diseases, 6 Sosnovy Blvd., Kemerovo, 650002, Russia; Head of Laboratory of Tissue Engineering and Intravascular Visualization Research Institute for Complex Issues in Cardiovascular Diseases, 6 Sosnovy Blvd., Kemerovo, 650002, Russia

**Keywords:** vascular patch, tissue engineering, silk fibroin, electrospinning, biocompatibility

## Abstract

**The aim of the study** was to make a vascular patch based on regenerated silk fibroin (SF) and study its physical and mechanical characteristics, biocompatibility and matrix properties in comparison with polyhydroxybutyrate/valerate/polycaprolactone with incorporated vascular endothelial growth factor (PHBV/PCL/VEGF) and commercial bovine xenopericardium (XP) flap in experiments *in vitro*.

**Materials and Methods:**

Tissue-engineered matrices were produced by electrospinning. The surface structure, physical and mechanical characteristics, hemocompatibility (erythrocyte hemolysis, aggregation, adhesion and activation of platelets after contact with the material) and matrix properties of vascular patches (adhesion, viability, metabolic activity of EA.hy926 cells on the material) were studied.

**Results:**

The surface of SF-based matrices and PHBV/PCL/VEGF-based tissue engineered patches had a porous and fibrous structure compared to a denser and more uniform XP flap. The physical and mechanical characteristics of SF matrices were close to those of native vessels. Along with this, tissue-engineered patches demonstrated high hemocompatible properties, which do not differ from those for commercial XP flap. Adhesion, viability, and metabolic activity of EA.hy926 endothelial cells also corresponded to the previously developed PHBV/PCL/VEGF matrix and XP flap, which indicates the nontoxicity and biocompatibility of SF matrices.

**Conclusion:**

Matrices produced from regenerated SF demonstrated satisfactory results, comparable to those for PHBV/PCL/VEGF and commercial XP flap, and in the case of platelet adhesion and activation, they outperformed these patches. In total, SF can be defined as material having sufficient biological compatibility, which makes it possible to consider a tissue-engineered matrix made from it as promising for implantation into the vascular wall.

## Introduction

One of the most common pathologies of the cardiovascular system is atherosclerosis. The formation of atherosclerotic plaques entails violation of blood vessel patency and deterioration in the blood supply of tissues and organs. Atherosclerosis of the internal carotid artery leads to carotid stenosis [1], which is the cause of ischemic stroke in 15% of cases [2].

One of the ways to restore blood flow in case of significant stenosis of the internal carotid artery (70% or more) is carotid endarterectomy [3]. In the presence of prolonged atherosclerotic plaques, carotid endarterectomy with closing the arteriotomy access using a vascular patch is preferable [4].

The material for a vascular patch can be of natural origin (bovine xenopericardium (XP), decellularized matrix, fibrin, collagen) or artificially synthesized (polyurethane, polyvinyl alcohol, polyethylene terephthalate, polycaprolactone). In addition, a promising scientific direction is the creation of vascular prostheses using tissue engineering methods [5]. This makes it possible to produce matrices with predetermined properties: structural stability and controlled biodegradation, recruitment of cells to colonize the prosthesis surface, and low immunogenicity [6].

One type of natural material is silk fibroin (SF), which is obtained from *Bombyx mori* silk fibers [7, 8]. In experiments *in vitro* and *in vivo*, SF demonstrates a high ability for adhesion, proliferation, and differentiation of stem cells [9], it is used to stimulate tissue regeneration [10-12], has low immunogenicity and antigenicity [13], and does not require stringent conditions for the production of biological matrices [14]. In addition, the works of recent years confirm the prospects of the development of SF products for the needs of cardiovascular surgery [15, 16].

**The aim of the study** was to produce a vascular patch based on regenerated silk fibroin and study its physical and mechanical characteristics, biocompatibility, and matrix properties in comparison with patches made of polyhydroxybutyrate/valerate/polycaprolactone with incorporated vascular endothelial growth factor and a bovine pericardial flap in experiments *in vitro*.

## Materials and Methods

The study was approved by the local Ethics Committee of the Research Institute for Complex Issues of Cardiovascular Diseases (Kemerovo, Russia), protocol No.6 of June 30, 2022.

### Producing vascular patches

Vascular patches obtained from 15% regenerated SF solution were produced by electrospinning on a NANON-01A device (MECC CO, Japan). Hexafluoropropanol was used as a solvent. The subsequent modification of the obtained matrices was performed in 98% ethanol in order to transfer SF from the regenerated (water-soluble) form to a water-insoluble one by forming β-links between protein molecules [17]. Electrospinning of SF matrices was carried out with the following parameters: a 22G needle; 15-cm distance to the collector; 20-kV voltage; collector rotation speed of 200 rpm; solution flow rate of 1 ml/h. A metal pin with a diameter of 8.0 mm was used as a collector.

Biodegradable patches with incorporated vascular endothelial growth factor (VEGF; Sigma-Aldrich, USA) were made by emulsion electrospinning from a mixture of 5% polyhydroxybutyrate/valerate (poly(3-hydroxybutyrate-co-3-hydroxyvalerate), PHBV; Sigma-Aldrich, USA) and polycaprolactone (poly(ε-caprolactone), PCL; Sigma-Aldrich, USA) in trichloromethane in a ratio of 1:2, mixing it with VEGF in saline (10 μg/ml) in a ratio of 20:1 [18]. The optimal parameters for electrospinning of PHBV/ PKL/VEGF patches were as follows: a 22G needle, 15-cm distance to the collector, 20-kV voltage, collector rotation speed of 200 rpm, solution flow rate of 0.5 ml/h. A metal pin with a diameter of 8.0 mm was used as a collector. The matrices were cut lengthwise and removed from the pin with gradual peeling movements.

The properties of tissue-engineered matrices were evaluated in comparison with flaps from the KemPeriplas-Neo bovine XP (NeoKor CJSC, Russia), which are used as a patch in vascular surgery during carotid endarterectomy.

### Study of the surface structure of patches

Specimens of vascular patches with a size of 0.25 сm^2^ were sprayed with Ag-Pd using an EM ACE200 system (Leica Microsystems GmbH, Austria) to obtain 15-nm thick coating. Structural features of the matrix surface were studied using an S-3400N scanning microscope (Hitachi, Japan) under high vacuum conditions at an accelerating voltage of 10 kV.

### Evaluation of physical and mechanical properties of patches

The cutting of all specimens was carried out in the longitudinal direction (n=5 in each group). The evaluation of the physical and mechanical properties of vascular patches was carried out in accordance with GOST 270-75 under uniaxial tension on a universal testing machine of the Z series (Zwick/Roell, Germany) using a sensor with a nominal force of 50 N; the traverse speed during testing was 50 mm/min. The strength of the material was determined from the maximum tensile stress of the specimens (MPa) and the force applied to the specimen before its destruction (Fmax (N)). The stress-related characteristics of the material were evaluated by relative elongation, corrected for the nature of the destruction of the specimens (it characterizes the elasticity of the material (%)), and Young’s modulus (it characterizes the stiffness of the material (MPa)).

The XP flaps were a comparison group. The sheep carotid artery (*a. carotis*) and the human internal thoracic artery (*a. mammaria*), which were obtained during coronary artery bypass surgery in patients who signed a voluntary informed consent to take the material, were used as controls.

### Evaluation of hemocompatibility of vascular patches

The hemocompatibility of tissue-engineered vascular patches was determined by the degree of erythrocyte hemolysis, aggregation, adhesion and activation of platelets after contact with the test material.

### Evaluation of the degree of erythrocyte hemolysis

The degree of erythrocyte hemolysis was determined after contact of the test material with fresh citrated blood. Specimens of patches (n=6) 25 cm^2^ in size were incubated in cuvettes with 10 ml of saline solution in a thermostat at 37°C for 2 h. Then, 200 μl of fresh citrated blood was added to each cuvette, mixed, and kept in a thermostat at 37°C for 60 min. Saline and distilled water were used as positive and negative controls, correspondingly. After completion of the incubation, the solution from the cuvettes was taken into test tubes and centrifuged at 2800 rpm for 10 min in order to precipitate erythrocytes. Optical density of supernatant solutions was measured on a Genesys 6 spectrophotometer (Thermo Fisher Scientific, USA) at a wavelength of 545 nm. The degree of hemolysis (*H* (%)) was determined by the formula (*Dt*–*Dne*)/(*Dpe*–*Dne*)**·**100%, where *Dt* is the optical density of the sample incubated with the test specimen; *Dne* is the optical density of the negative control (saline probe); *Dpe* is the optical density of the positive control (samples after 100% hemolysis) [19, 20]. The average value of optical density when measuring samples of saline with blood (it was equal to 0) was taken as a positive control (complete absence of hemolysis). The average value of the optical density of samples after incubation of blood with distilled water (100% hemolysis) was taken as a negative control.

### Assessment of platelet aggregation

Platelet aggregation was assessed after contact of donor plasma with the test material in accordance with ISO 10993.4. A 3.8% sodium citrate solution (9:1 ratio) was added to fresh donated blood and then centrifuged at 1000 rpm for 10 min. The resulting platelet-rich plasma (PRP) was used as a positive control for the platelet aggregation reaction. To calibrate the device, platelet-poor plasma was used, which was obtained as a result of repeated PRP centrifugation at 4000 rpm for 20 min. The test specimens were placed in cuvettes with PRP for 3 min, then platelet aggregation inducer ADP (AGRENAM, AG-6; Research and Production Association RENAM, Russia) was added at a concentration of 20 μM/l. Platelet aggregation was assessed using a semi-automatic 4-channel analyzer ARAST 4004 (LABiTec, Germany). After 5 min, the maximum percentage of platelet aggregation (%) was recorded.

### Evaluation of platelet adhesion

The degree of platelet adhesion was determined after incubation of test specimens 0.25 cm^2^ in size with 300 μl of PRP for 1 h at 37°C. In order to remove non-adherent platelets, the products were washed in phosphate-buffered saline (PBS; pH 7.4), then fixed in 4% paraformaldehyde solution for 10 min. Next, the specimens were incubated with rabbit antibodies to CD41 (ab134131; Abcam, UK) and mouse antibodies to CD62P (ab54427; Abcam, UK) for 12 h at 4°C. After that, the matrices were washed with PBS with the addition of 0.1% Tween 20. Then, the specimens were incubated for 1 h at room temperature with goat secondary antibodies to rabbit IgG conjugated with Alexa Fluor 488 (A11034; Thermo Fisher Scientific, USA) and goat antibodies to mouse IgG conjugated with Alexa Fluor 555 (A31570; Thermo Fisher Scientific, USA). The sections were repeatedly washed with PBS with the addition of 0.1% Tween 20. A confocal microscope (LSM700; Carl Zeiss, Germany) was used to analyze the products.

### Study of matrix properties

The adhesive properties of matrices were studied by the area occupied by the Talin focal adhesion protein. For this purpose, the prepared specimens were fixed for 10 min in a 4% paraformaldehyde solution and permeabilized with 0.1% Triton X100 for 15 min. Non-specific binding was blocked with 1% bovine serum albumin in PBS for 1 h at room temperature. Next, the specimens were incubated with Talin rabbit primary antibodies (abcam, ab71333; Abcam, UK) at 4°C overnight. After washing in PBS, the specimens were incubated for 1.5 h with Donkey anti-Rabbit IgG (H+L) Highly Cross-Adsorbed Secondary Antibody Alexa Fluor 488 (A21206; Thermo Fisher Scientific, USA) and Phalloidin Alexa Fluor 568 (A12380; Invitrogen, USA). Then, the specimens were washed again with PBS to remove unbound secondary antibodies, and the nuclei were stained with 10 mg/ml DAPI (4’,6-diamidino-2-phenylindole dihydrochloride) (D9542; Sigma-Aldrich, USA) for 40 min. The finished specimens were washed and mounted under coverslips in a ProLong medium (P36930, Life Technologies, USA) and examined on an LSM 700 confocal laser scanning microscope (Carl Zeiss, Germany).

### Statistical data processing

Statistical data processing was performed using the Prism program (Graph Pad Software, USA). The correspondence of the distribution of the obtained data to the normal one was assessed using the Kolmogorov–Smirnov test. Two independent groups were compared using the Mann– Whitney test. To assess intergroup differences in three or more groups, the nonparametric Kruskal–Wallis test was used; the Dunn test was used for pairwise comparison of groups. Differences were considered significant at a significance level of p<0.05, with pairwise comparison of groups it was p<0.05/k, where k is the number of compared groups. The data were presented as median, 25^th^ and 75^th^ percentiles (Me [25%; 75%]).

## Results and Discussion

### Structural features of vascular patches

**Figure 1.**
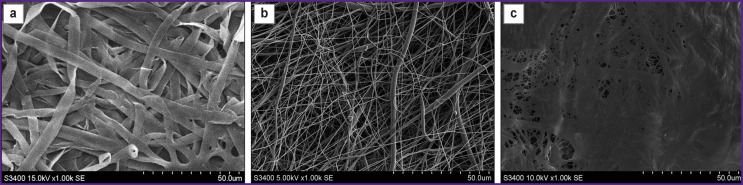
Ultrastructure of materials: (a) the inner surface of the silk fibroin matrix; (b) the inner surface of a polyhydroxybutyrate/valerate/polycaprolactone matrix with incorporated vascular endothelial growth factor (PHBV/PCL/VEGF); (c) serous side of a flap from bovine xenopericardium. Scanning electron microscopy, ×1000

Scanning electron microscopy of SF specimens showed that their inner surface is represented by closely intertwined flat fibers; there are areas of fiber adhesions, as well as a small number of shallow pores (Figure 1 (a)). The average fiber diameter on the surface was 4.80±1.39 μm.

The surface of PHBV/PCL/VEGF matrices has also been found to have a highly porous fibrous structure represented by multidirectional fibrils 1.47±0.67-μm thick (Figure 1 (b)).

Scanning electron microscopy of XP flaps has shown the preservation of their native structure; the surface is relief due to crimped collagen fibers. The presence of single pores on the surface can be explained by the dense arrangement of collagen fibrils (Figure 1 (c)).

Thus, the SF and PHBV/PCL/VEGF specimens had a more “loose” and porous structure compared to the XP flap. This feature of the ultrastructure of tissue-engineered matrices may offer an advantage when they are colonized by endothelial cells after implantation into the vascular wall [21].

### Physical and mechanical characteristics of vascular patches

**Figure 2.**
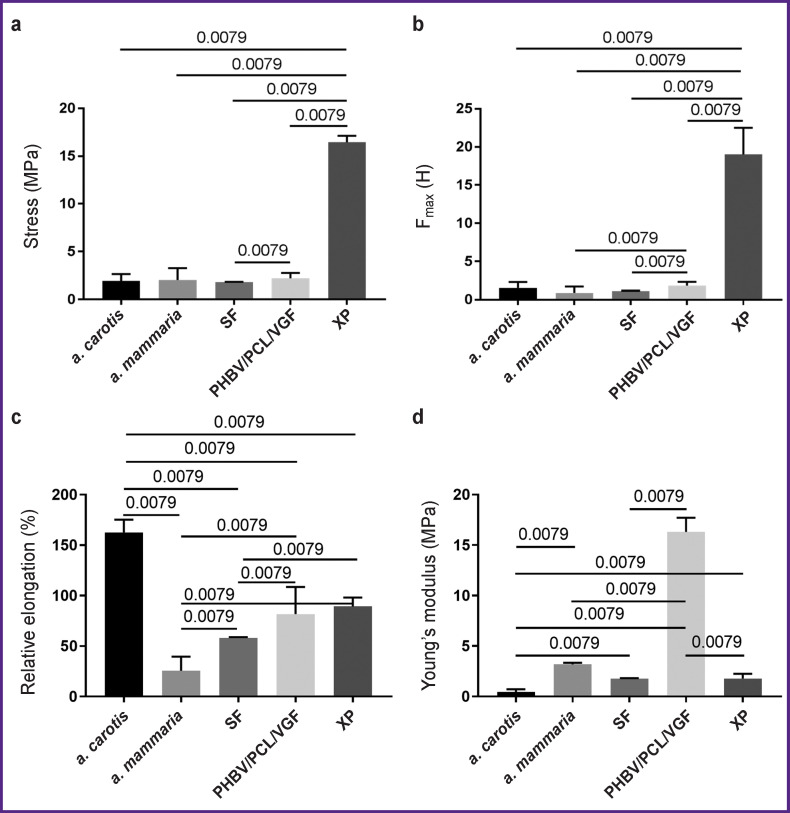
Physical and mechanical characteristics of tissue-engineered vascular patches: (a) stress, characterizing the material strength; (b) F_max_ is the force required to break the material; (c) relative elongation, characterizing the material elasticity; (d) Young’s modulus, characterizing the material stiffness. SF is silk fibroin, XP is bovine xenopericardium, PHBV/PCL/VGF is polyhydroxybutyrate/valerate/polycaprolactone with incorporated vascular endothelial growth factor

SF patches are similar in strength to native vessels — human *a. mammaria* and sheep *a. carotis* (p<0.01) (Figure 2 (a), (b)). The elasticity of SF matrices was 2.8-fold lower than that of the carotid artery, but it was 2.3-fold higher than the elasticity of the internal mammary artery (p<0.01) (Figure 2 (c)). The stiffness of SF patches ranged between the values of this indicator in native vessels (p<0.01) (Figure 2 (d)). Probably, such physical and mechanical characteristics of SF are due to the features of its semicrystalline ultrastructure [22].

The durability of the PHBV/PCL/ VEGF matrices corresponded to that of sheep *a. carotis* and human *a. mammaria* (see Figure 2 (a)). However, the force applied to the specimen prior to its destruction was 2-fold higher for the PHBV/ PCL/VEGF patches than for the internal mammary artery (p<0.01) (see Figure 2 (b)). PHBV/PCL/VEGF matrices were almost 2-fold less elastic than *a. carotis* (p<0.01), and 3-fold more extensible than *a. mammaria* (p<0.01) (see Figure 2 (c)). At the same time, the stiffness of the PHBV/PCL/VEGF material was the highest among all the specimens (see Figure 2 (d)).

Physical and mechanical characteristics of XP flaps differed from those of native vessels (see Figure 2). The durability and tear resistance of XP was the highest among all the studied materials (see Figure 2 (a), (b)). Similar results were obtained when assessing the durability of biological patches in cardiac surgery [23]. The elasticity of the XP flaps was almost 2-fold lower than that of the sheep carotid artery and 3.5-fold higher than that of the human internal mammary artery (p<0.01) (see Figure 2 (c)). The stiffness of the XP flap was 9-fold lower than that of the PHBV/PCL/VEGF patches (p<0.01) (see Figure 2 (d)).

Generally, physical and mechanical tests have shown that SF matrices are closest in their characteristics to the properties of native arteries, which contributes to adequate integration of the material in further implantation of patches into the vascular wall.

### The results of the evaluation of erythrocyte hemolysis

The degree of erythrocyte hemolysis after blood contact with SF and PHBV/PCL/VEGF matrices was insignificant and did not differ significantly between the groups (see the Table), which indicates a high hemocompatibility of the studied materials [24].

**Table:** The results of hemolysis and platelet aggregation (n=6 in each group), Me [25%; 75%]

Specimen	Degree of erythrocyte hemolysis (%)	Maximum of platelet aggregation (%)
Silk fibroin	0.006 [0.004; 0.024]	86.41 [84.45; 90.03]
PHBV/PCL/VEGF	0.002 [0.001; 0.002]	86.02 [83.02; 87.62]
Bovine xenopericardium	0.020 [0.016; 0.025]*	85.03 [83.57; 87.16]
Platelet-rich plasma	—	83.97 [81.75; 86.14]

* p<0.017 compared to PHBV/PCL/VEGF (polyhydroxybutyrate/ valerate/polycaprolactone with incorporated vascular endothelial growth factor).

Hemolysis of erythrocytes after contact with the XP flap was higher than after contact with SF and PHBV/ PCL/VEGF — 3.3- and 10.0-fold, respectively (p<0.017) (see the Table), but did not exceed the maximum allowable values [24].

### The results of the assessment of platelet aggregation

After plasma contact with the test specimens, no statistically significant intergroup differences in platelet aggregation were found. All materials caused insignificant platelet aggregation, which did not differ from that in PRP (see the Table).

### Results of evaluation of adhesion and activation of platelets

**Figure 3.**
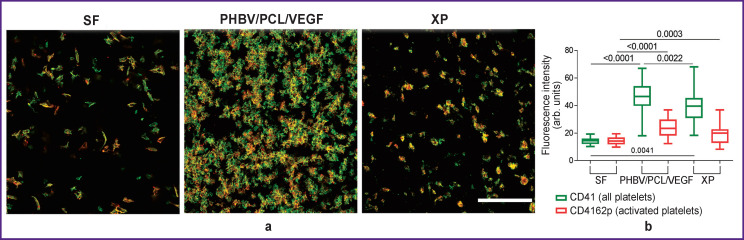
Confocal microscopy of platelets: green — CD41; red — CD62p; yellow — color overlay, bar — 50 μm (a), and signal intensity of CD41 and CD62p (b) SF is silk fibroin, XP is bovine xenopericardium, PHBV/PCL/VEGF is polyhydroxybutyrate/valerate/polycaprolactone with incorporated vascular endothelial growth factor

The least platelet adhesion was recorded on SF matrices. A similar pattern was observed for XP flaps. Platelet adhesion was more pronounced on PHBV/PCL/VEGF patches (Figure 3 (a)). Along with this, the least activation of adherent platelets was observed on SF matrices, the level of which was statistically significantly different from that for PHBV/PCL/VEGF matrices and XP flaps (p<0.017) (Figure 3 (b)).

Based on the data obtained, it can be concluded that the SF matrices have optimal hemocompatibility, which corresponds to that for PHBV/PCL/VEGF- and XP-based patches, and in the case of platelet adhesion and activation, the hemocompatibility of SF specimens even outperforms other products.

### Results of evaluation of matrix properties of vascular patches

**Figure 4.**
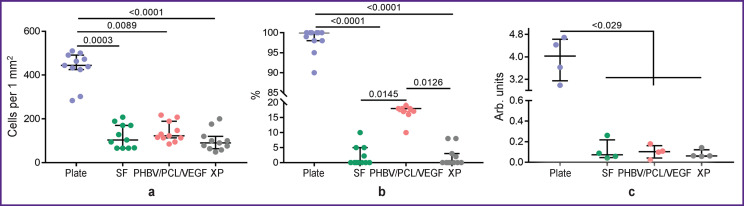
Matrix properties of vascular patches: (a) cell density per 1 mm^2^; (b) viability; (c) metabolic activity. SF is silk fibroin, XP is bovine xenopericardium, PHBV/PCL/VEGF is polyhydroxybutyrate/valerate/polycaprolactone with incorporated vascular endothelial growth factor

Cultivation of EA.hy926 cells on the surface of the studied matrices for 3 days showed identical biological attractiveness of all studied materials. Thus, the total number of adherent cells on all types of matrices, on average by medians, was at the level of 105.7 cells/mm^2^, which is 4-fold lower than the colonization density of EA.hy926 on the cultural plate — 444.2 [425.3; 491.4] cells/mm^2^, p<0.05 (Figure 4 (a)). The viability of endothelial cells did not reach high values either and varied from 0 to 33% (Figure 4 (b)). Viability is inseparable from the metabolic activity of cells, which was kept at an equal level for all types of matrices — 0.1 arb. unit (Figure 4 (c)).

**Figure 5.**
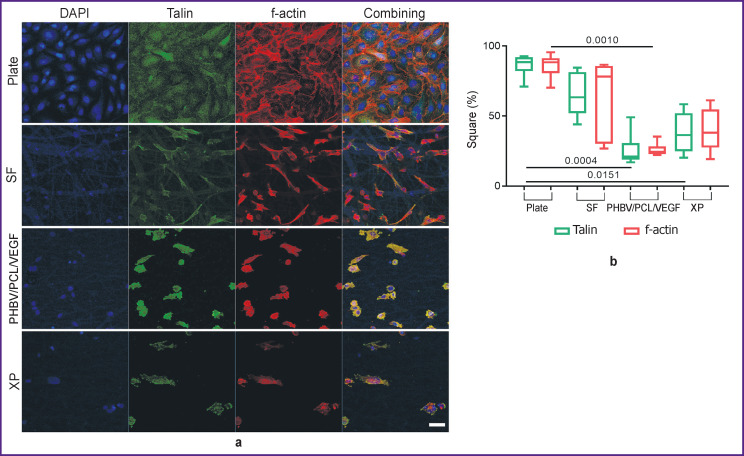
Confocal microscopy of EA.hy926 cells: blue — DAPI; green — Talin; red — f-actin, bar — 50 μm (a), and Talin and f-actin area (b) SF is silk fibroin, XP is bovine xenopericardium, PHBV/PCL/VEGF is polyhydroxybutyrate/valerate/polycaprolactone with incorporated vascular endothelial growth factor

Immunofluorescent staining of endothelial cells for the focal adhesion protein Talin and the cytoskeletal protein f-actin showed the features of the landscape of each studied material (Figure 5 (a)). On the cultural plate, a reference monolayer of cells was obtained, which tended to their regular hexagonal shape and were in close contact with each other. A similar picture is observed in the early stages of endothelialization of vascular prostheses [25].

The area of focal adhesion of the Talin protein for the cultural plate was the largest one and corresponded to the density of the cytoskeletal protein, f-actin (Figure 5 (b)). No monolayer of cells was found on all types of matrices. On SF, the activation of integrin receptors and the formation of foci of focal adhesion were maximal in comparison with other matrices: the largest area of Talin and f-actin was noted. On the SF surface, foci of accumulation of spindle-shaped cells with a small number of contacts were found; however, at the same time, conglomerates of snarled cells were present. On PHBV/PCL/VEGF and XP, the shape and pattern of distribution of fluorescent proteins were similar: from round to more evenly elongated in all directions. There were slightly more intercellular contacts on PHBV/PCL/ VEGF (probably due to the isolation of the vascular growth factor from the polymer filaments).

On the whole, it can be concluded that the matrix properties and adhesiveness of the new developed material based on 15% SF are at the level of the previously developed artificial PHBV/PCL/VEGF material and decellularized commercial bovine XP.

## Conclusion

In the present study, a comprehensive comparative evaluation of the physical and mechanical characteristics and biocompatibility of tissue-engineered vascular patches was performed in experiments *in vitro.* For most of the parameters studied, matrices produced from 15% silk fibroin demonstrated satisfactory results, comparable with those for the previously developed PHBV/PCL/ VEGF material, as well as the commercial XP flap, and in the case of platelet adhesion and activation, they outperformed the indicated patches.

In total, silk fibroin can be defined as a material with sufficient biological compatibility, which makes it possible to consider a tissue-engineered matrix produced from it as promising for implantation into the wall of blood vessels. However, further research of these matrices as vascular patches is needed.
